# Comparison of the accuracy of two guiding devices for femoral tunnel position in anterior cruciate ligament reconstruction surgery: A radiographic study

**DOI:** 10.1002/jeo2.70632

**Published:** 2026-01-19

**Authors:** Pei Zhao, Shixin Nie, Zhi Chen, Chengjian Wu, Jiajun Lin, Chengjie Lian, Hua Zhang

**Affiliations:** ^1^ Department of Orthopaedics, Sports Injury Division Fujian Medical University Union Hospital Fuzhou China

**Keywords:** anterior cruciate ligament reconstruction, femoral locator, femoral tunnel position, I.D.E.A.L.

## Abstract

**Purpose:**

This study evaluated the comparative efficacy of two guiding devices in positioning the femoral tunnel in the ‘Isometric, Direct insertion, Eccentrically located, Anatomical, Low tension’ (I.D.E.A.L.) zone during anterior cruciate ligament reconstruction.

**Methods:**

A retrospective analysis was conducted on 64 patients. Group A (32 cases) used traditional tools, while Group B (32 cases) used the modified locator. Postoperative three‐dimensional computed tomography reconstructions of the femoral condyle were performed, and tunnel position (using the quadrant method) and the distance from the tunnel's posterior edge to the lateral condyle posterior wall were measured. Statistical analysis was performed to compare the differences between the two groups.

**Results:**

The mean femoral tunnel centre positions were 30.3% ± 4.5% (deep–shallow) and 25.5% ± 9.6% (high–low) in Group A, and 25.9% ± 3.9% (deep–shallow) and 26.6% ± 5.8% (high–low) in Group B. The ideal I.D.E.A.L. tunnel centre was defined at (28.4, 22.2). No significant difference in dispersion was observed between the two groups. But the tunnel centre in Group B is more concentrated near the posterior region of the femoral footprint. The distance from the posterior tunnel edge to the posterior wall was 3.5 ± 1.7 mm in Group A and 2.2 ± 1.0 mm in Group B, showing a significant difference. Each group had 1 case of the posterior wall rupture; the rupture rate did not differ significantly between the groups.

**Conclusion:**

The modified locator facilitates femoral tunnel placement closer to the posterior wall, which promotes the biomechanical conditions required for optimal graft isometry and low tension.

**Level of Evidence:**

Level IV and V, retrospective study.

**Abbreviations**
ADC: apex of the deep cartilage
D‐S: deep‐to‐shallow
H‐L: high‐to‐low
I.D.E.A.L.: Isometric properties, Direct insertion fibres, Eccentrically located positioning, Anatomical footprint, Low tension

## INTRODUCTION

Anterior cruciate ligament (ACL) reconstruction is the mainstream surgical intervention for ACL‐injured patients aiming to return to high‐level physical activities. Despite excellent clinical outcomes after ACL reconstruction, patients with high athletic demands continue to experience a relatively high postoperative failure rate [24, 25]. Approximately 50% of technical failures are attributed to improper femoral tunnel positioning [18, 22]. Notably, Ziegler et al. [29] reported that almost 95% of revision ACL surgeries involve incorrect tunnel positions. Therefore, accurately locating the femoral insertion footprint and establishing a bone tunnel accordingly are critical for surgical success. The optimal femoral tunnel position, however, remains controversial. Following Freddie Fu's proposal of the anatomical reconstruction concept [21, 27], the anatomical centre of the femoral footprint was regarded as the ideal tunnel location [4, 6, 13] in a long time with numerous studies reporting good clinical outcomes. However, a potential limitation of anatomical footprint‐centred ACL reconstruction is the inherent challenge in achieving ideal graft isometry [12, 15]. Moreover, grafts positioned at the anatomical centre are subjected to elevated loads, thereby increasing the risk of postoperative failure [1, 17]. In 2015, Pearle et al. [14] introduced the concept of the femoral Isometric, Direct insertion, Eccentrically located, Anatomical, Low tension (I.D.E.A.L.) position. As this region offers potential isometry and reduced graft tension, some surgeons may choose this area as their preferred tunnel location [11, 23].

The I.D.E.A.L. concept refers to a femoral tunnel position that should exhibit *isometric* properties (good length‐change behaviour throughout knee motion); coverage of the ACL's *direct insertion* fibres; *eccentrically located* positioning in the anterior‐superior (high and deep) region of the footprint; placement within the *anatomical* footprint; and *low tension* on the graft during knee flexion‐extension [14]. To achieve this posterior‐superior tunnel position intraoperatively, surgeons typically use the apex of the deep cartilage (ADC) of the lateral femoral condyle as a landmark. The locator is positioned flush against the ADC, with the tunnel centre offset 5–7 mm anteriorly from this cartilage edge. However, the short ‘tip’ of traditional locators may at times achieve only suboptimal contact with the posterior condylar wall. This incomplete adaptation can compromise tunnel positioning accuracy. To overcome this limitation and ensure consistent precision, a modified device has been developed to address this. The ‘tip’ of the locator was extended and modified into a curved shape to ensure better alignment and contact with the posterior wall of the lateral femoral condyle during use. Additionally, the range of eccentric offset distances was increased (5–13 mm). Through these modifications, surgeons are better assisted in preparing the femoral tunnel during surgery. This allows the posterior edge of the tunnel aperture to be positioned closer to the posterior wall of the lateral femoral condyle (<2 mm) and enables the tunnel to be located more precisely within the I.D.E.A.L. zone.

We hypothesise that this instrument allows for superior tunnel preparation, thereby improving reconstruction biomechanics. This study thus aims to retrospectively compare the accuracy of femoral tunnel positioning between this novel device and the conventional one.

### Methods

The study was conducted in accordance with the Declaration of Helsinki, and the protocol was approved by the Ethics Committee of the Fujian Medical University Union Hospital (approval number [2025KY686]). A retrospective analysis was conducted on patients who underwent ACL reconstruction at our institution between April 2024 and September 2025. The inclusion criteria were defined as follows: (1) single‐bundle ACL reconstruction in which the femoral tunnel was prepared using the I.D.E.A.L. positioning method; (2) complete computed tomography (CT) imaging data obtained within one week postoperatively; and (3) documented and traceable use of positioning instruments, as verified by surgical records or intraoperative images. The exclusion criteria were applied as follows: (1) concurrent bony procedures, such as femoral or tibial osteotomy, were performed; and (2) femoral‐sided ACL avulsion fractures were present.

The sample size was estimated using G*Power software (version 3.1.9.7). A two‐tailed independent‐samples *t* test was selected to compare differences between the experimental and control groups, with an allocation ratio of 1:1. Based on preliminary data, the expected effect size (Cohen's d) was set at 0.80. The Type I error rate (α) was set at 0.05, and the Type II error rate (β) was set at 0.20, corresponding to a statistical power of 80%. The power analysis indicated that at least 26 participants per group (52 in total) would be required to achieve the desired power.

A total of 64 patients were included in this study. Based on the femoral locator used during surgery, patients were divided into Group A (conventional, offset‐type femoral locator) and Group B (modified locator), Each group consisted of 32 participants. Analysis of baseline characteristics showed no significant differences between the two groups (Table 1).

**Table 1:** Patient demographic data.

Items	Group1 (n = 32)	Group2 (*n* = 32)
Age (years)	34.5 ± 12.8	28.3 ± 10.3
Gender (F/M)	12/20	15/17
Hight (cm)	167.1 ± 7.0	169.6 ± 7.4
Weight (kg)	68.1 ± 12.1	68.2 ± 9.9
BMI (kg/m^2^)	24.3 ± 3.6	23.6 ± 2.7

Abbreviation: BMI, body mass index.

**Figure 1:**
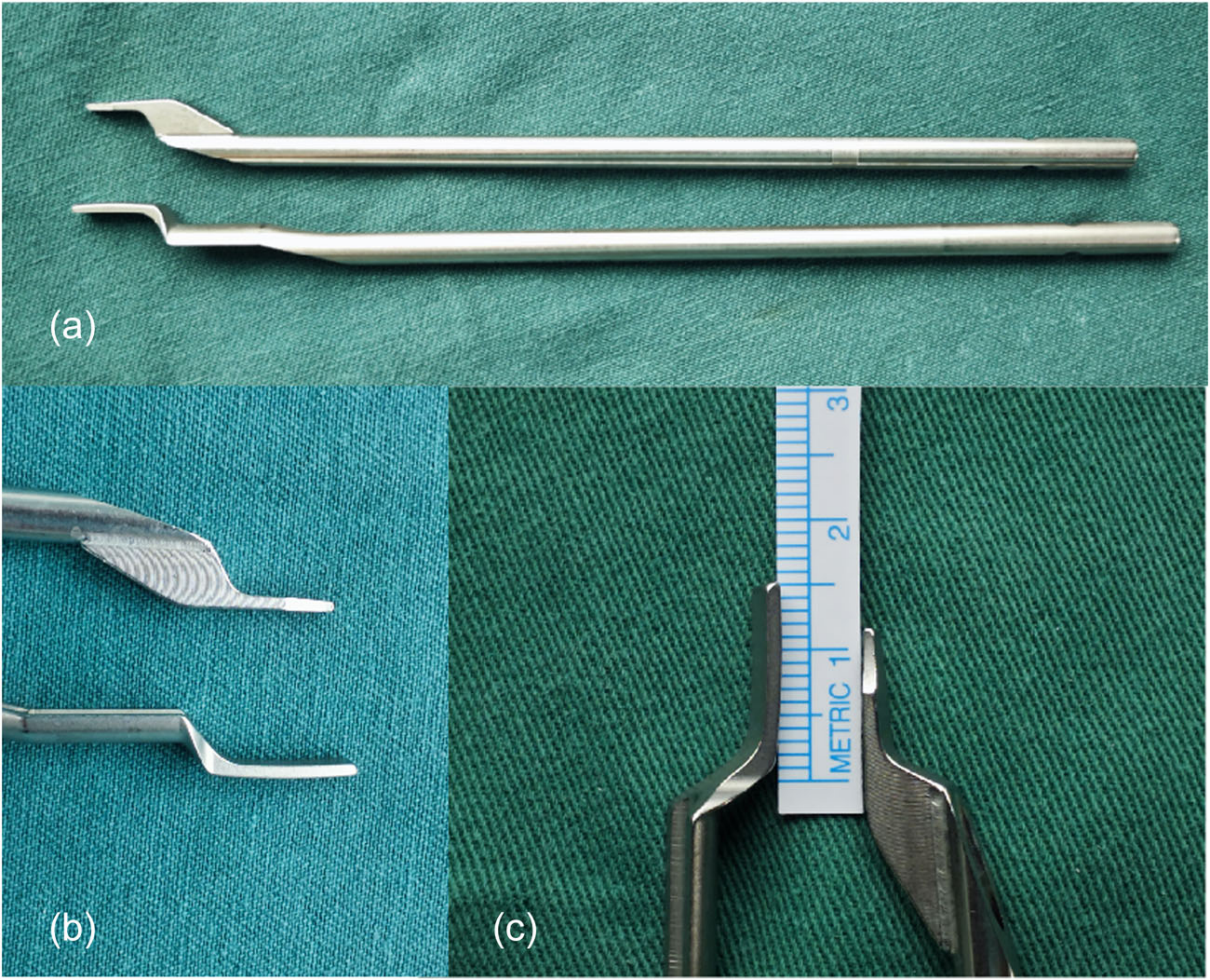
(a) Conventional locator (upper) and modified locator (lower). (b) Curved surface modification of the tip in the modified locator (lower). (c) Modified locator (left) with longer tip length.

The modified locator was designed with several key improvements over traditional tools. The ‘tip’ length was extended from 10 mm to 15 mm; its contact surface was curved to better conform to the posterior femoral condyle (Figure 1); and the range of offset distances was expanded to offer incremental options from 5 mm to 13 mm. This enables more reliable contact to be achieved and precise tunnel positioning to be maintained. The expanded offset range is intended to accommodate various approaches, utilising 5–7 mm for the I.D.E.A.L. zone and 10–13 mm for the anatomical centre.

### Surgical technique

The patient is positioned supine under general anaesthesia. An anterolateral portal was established for assessing the intra‐articular structures and confirming the ACL injury. Then, a transpatellar tendon portal was created to facilitate the initial notch debridement. The arthroscope was then positioned through this portal, thereby providing an improved view for identifying the femoral ACL footprint. Under arthroscopic guidance, an anterior medial working portal is created. With the knee flexed to 60°–90°, the ACL remnant is debrided to fully expose the femoral footprint and the superior border of the posterior femoral condylar cartilage. The femoral footprint is carefully identified, and the I.D.E.A.L. zone is localised and marked using a radiofrequency (BONSS BDS313) device. The optimal I.D.E.A.L. tunnel position requires the superior border to closely approximate but not extend above the resident's ridge, while maintaining a posterior margin within 2 mm of the posterior femoral condylar wall [14]. Under visualisation through the central portal, the femoral tunnel is prepared via the anteromedial portal using the appropriate locator: First, an eccentric distance matching the graft diameter (with an offset 1–2 mm greater than the graft radius) is selected. The ‘tip’ of the eccentric locator is positioned at the ADC border of the posterior femoral condyle, with its midline aligned to the posterior cartilage edge and firmly opposed to the posterior wall of the lateral femoral condyle. After guide positioning, a guide pin is drilled to create an approximately 2 mm deep starting groove. While progressively increasing knee flexion, the guide pin position is maintained to ensure the pin tip remains within the starting groove. At maximum flexion (100°–120°), the guide pin is fully advanced, followed by sequential drilling of pilot (diameter 4.5 mm) and final tunnels (Do to graft diameter).

### Radiological measurement

**Figure 2:**
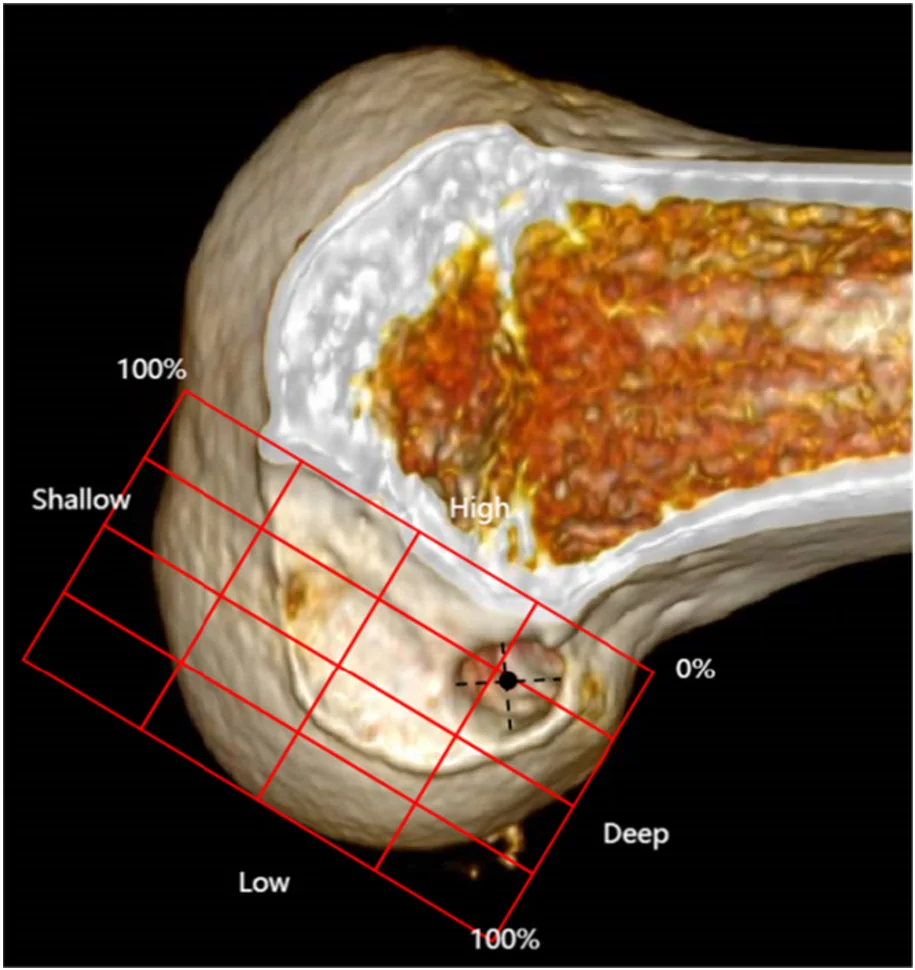
Tunnel centre measurement using the quadrant method.

Postoperative CT scans were performed using to reconstruct the distal femur. Initial evaluation was conducted in the lateral‐to‐medial view, with the imaging plane adjusted until the posterior aspects of the medial and lateral femoral condyles were perfectly superimposed. The image was then internally rotated by 90° to obtain a distal‐to‐proximal view, and the lowest point of the femoral trochlea was identified. A vertical cut was made at this reference point, preserving the lateral femoral condyle. The image was further internally rotated by another 90° to obtain a standardised medial view of the lateral femoral condyle. Tunnel position analysis was performed using the quadrant method: [7, 10] (1) a four‐grid coordinate system was constructed to measure the Deep‐to‐Shallow (D‐S) and High‐to‐Low (H‐L) dimensions. (2) The tunnel centre was defined as the intersection point between the tunnel's major axis and the longitudinal axis of the condyle. (3) The position of the tunnel centre was then calculated as a percentage along the D–S and H–L axes (Figure 2). The average values of all the data measured by two observers were entered into SPSS software (version 21.0; IBM Corp), and another orthopaedic surgeon conducted all the analysis independently. The interclass correlation coefficient (ICC) was calculated to identify the reliability of each parameter, with a value of >0.75 indicating excellent agreement. As depicted previously, the anatomic parameters were measured via the currently used software on CT images [7], which provided measurement accuracy to one decimal point in this study.

**Figure 3:**
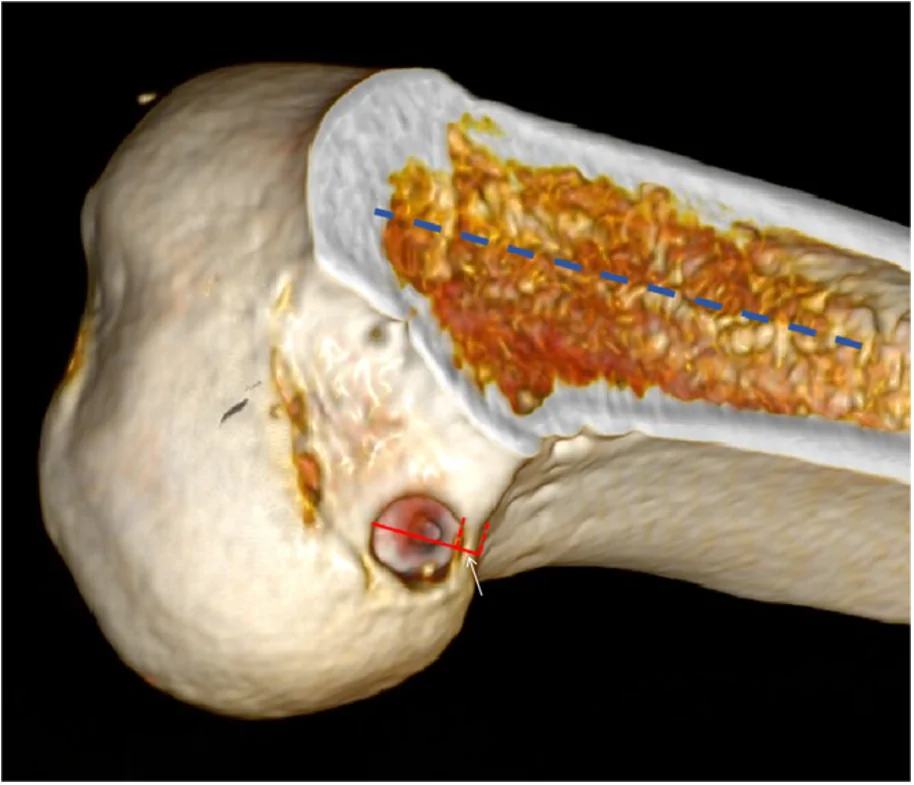
View with the tunnel entrance forms a complete circle and the posterior contour of the lateral femoral condyle appeared as a single continuous curve. Blue dotted line: the femoral shaft axis. White arrow: distance from the tunnel posterior edge to the lateral condyle posterior wall.

Distance from tunnel posterior edge to lateral condyle posterior wall: The standardised medial view of the lateral femoral condyle was reconstructed with the femoral shaft aligned parallel to the horizontal axis. The image was externally rotated along the vertical axis until the posterior contour of the lateral femoral condyle appeared as a single continuous curve, then adjusted rotation until the tunnel entrance forms a complete circle, corresponding to the arthroscopic viewing perspective. On this view, the centre of the femoral tunnel was identified, draw a line parallel to the femoral shaft axis was drawn through the centre. The distance between the intersection points of this line and the posterior bone cortex is defined as the posterior tunnel edge‐to‐wall distance (Figure 3).

### Statistical analysis

Statistical analysis was performed using SPSS software. Continuous data were expressed as mean ± standard deviation and analysed using *t* tests or rank‐sum tests depending on whether the data followed a normal distribution, while categorical data were evaluated using chi‐square tests.

## RESULT

### Femoral tunnel centre position

**Figure 4:**
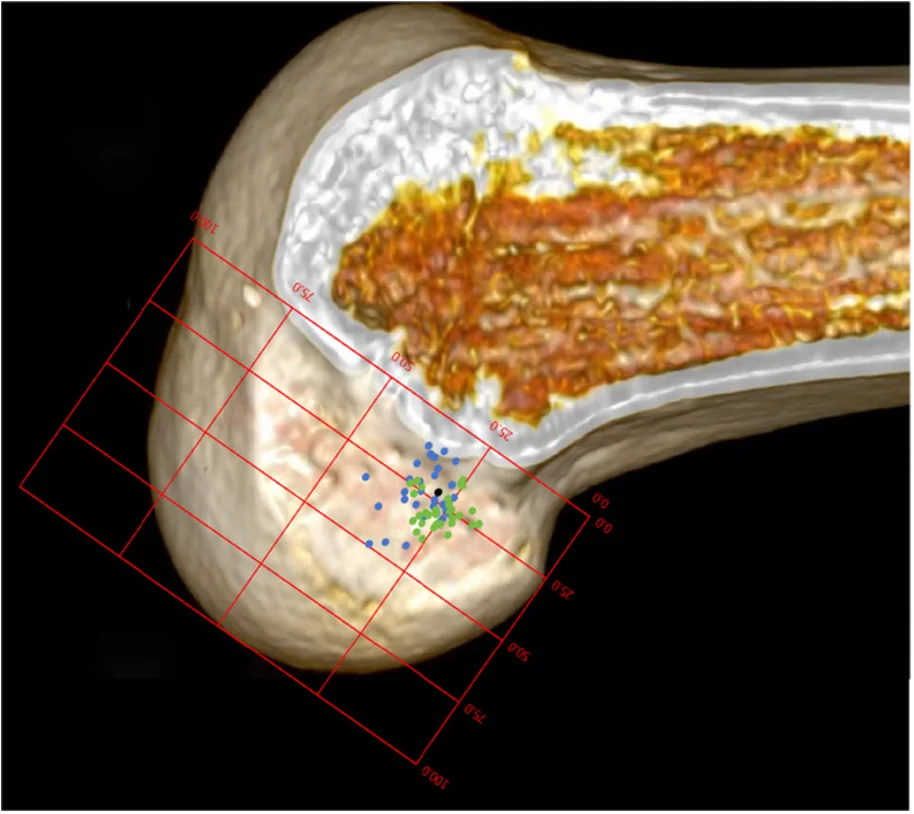
The tunnels' centre is shown. The blue scatter points represent the distribution of Group A, while the green scatter points represent the distribution of Group B, the black point is the reference I.D.E.A.L. point (28.4%, 22.2%). The distribution trend demonstrates that Group B's centre points cluster closer to the posterior region.

Using the quadrant method, the mean tunnel centre position in Group A was measured at 30.3 ± 4.5% (deep–shallow) and 25.5 ± 9.6% (high–low), while Group B showed mean positions of 25.9 ± 3.9% (deep–shallow) and 26.6 ± 5.8% (high–low). Based on previous research by Yin et al. [19, 28], the ideal I.D.E.A.L. tunnel centre is located at 28.4%, 22.2%. Using this reference point, we calculated the Euclidean distance from each coordinate to this ideal position as a dispersion value. Comparative analysis revealed no significant difference in dispersion between the two groups (Table 2), but in Group B, tunnels' centre demonstrated a distinct posterior positioning trend (Figure 4).

**Table 2:** Tunnel centre position by quadrant method.

	Group		*p* Value
	Group A(*n* = 32)	Group B(*n* = 32)	
Deep‐shallow	30.3 ± 4.5%	25.9 ± 3.9%	
High‐low	25.5 ± 9.6%	26.6 ± 5.8%	
Dispersion value^a^	7.9 (4.7, 11.5)	7.6 (5.2, 9.9)	0.7

^a^ The data were expressed as the median(interquartile) and were analysed using the Mann–Whitney *U* test due to non‐normality.

### Distance from posterior tunnel edge to posterior wall

Group A measured 3.5 ± 1.7 mm, while Group B showed 2.2 ± 1.0 mm, demonstrating a significant difference between the two groups (Table 3).

**Table 3:** Distance from posterior tunnel edge to femoral posterior wall.

	Group		*p* Value
	Group A(*n* = 32)	Group B(*n* = 32)	
Posterior wall distance	3.51 ± 1.7	2.20 ± 1.0	0.0

### Posterior wall breach

Cases of posterior wall breach occurred in both groups; each group had 1 case of breach. Chi‐square test analysis revealed no significant difference in the incidence of posterior wall breach between the two groups (Table 4).

**Table 4:** Posterior wall breach.

	Group (%)		Total	*p* Value
	Group A	Group B		
Posterior wall integrity	31 (96.9)	31 (96.9)	62 (96.9)	1.0
Posterior wall breach	1 (3.1)	1 (3.1)	2 (3.1)	
Total	32	32	64	

## DISCUSSION

The most important finding of the present study was that the use of the modified instrument enabled the creation of femoral tunnels that were located significantly closer to the posterior wall, without an increased risk of posterior wall breach. The advantage of the modified tool was confirmed through measurements: the posterior tunnel edge to posterior wall distance averaged 2.2 ± 1.0 mm with the modified instrument, compared to 3.5 ± 1.7 mm with the traditional instrument (*p*<0.01), indicating more consistent I.D.E.A.L. zone coverage. Notably, despite achieving closer posterior wall proximity, the modified device did not increase the risk of posterior wall breach (Group A: 3.1% vs. Group B: 3.1%, *p*=1.000). Since the introduction of the I.D.E.A.L. concept for ACL femoral tunnels by Pearle in 2015, optimal positioning has been defined by the following criteria: isometric behaviour, direct fibre incorporation, eccentric placement within the anatomical footprint, and low graft tension. This zone is located in the posterosuperior area of the ACL femoral footprint, with the ideal tunnel positioned in close proximity to the resident's ridge and with its posterior margin within 2 mm of the femoral posterior wall [15, 28]. It was found that the use of modified instruments enabled optimal tunnel placement within this target area. In this area, improved graft isometry and lower tension characteristics can be obtained. Although the native ACL is not completely isometric, it is difficult to restore the true footprint coverage and fibre orientation of the ACL during reconstruction. Therefore, it is suggested that placing the graft in a more isometric and low‐tension environment may be more suitable for the requirements of a reconstructed graft.

However, compromise may be made by surgeons due to the potential increase in posterior wall breach risk associated with closer tunnel placement to the posterior wall. Imperfect wall contact with traditional instruments may lead surgeons to adjust the tunnel centre anteriorly to avoid posterior wall breach, whereas the elongated, curved tail of the modified design was shown to maintain anatomical contact without compromising safety. To accommodate diverse surgical needs, a graduated series of offset options (ranging from 5 mm to 13 mm in 1 mm increments) was engineered. The 5–7 mm offset range was found to optimise I.D.E.A.L. tunnel placement, while the 10–13 mm offsets facilitated anatomical centre reconstruction.

The study demonstrated that accurate placement of the ACL femoral tunnel within the target zone was achieved with both types of instruments. Satisfactory femoral tunnel positioning was achieved with both the traditional and modified locators. In the traditional tool group, the mean coordinates were 30.3% ± 4.5% (deep–shallow) and 25.5% ± 9.6% (high–low), whereas in the modified locator group, the coordinates were 25.9% ± 3.9% (deep–shallow) and 26.6% ± 5.8% (high–low). Scatter plot analysis revealed that most tunnel centres in both groups were clustered within the posterosuperior area of the femoral footprint. The quadrant method is widely utilised in clinical studies of ACL insertion sites [8, 16, 19, 20]. Initially applied on standard lateral radiographs [2], its application was later extended to CT scans [3, 5, 9] and its reliability has been confirmed, though considerable variability is observed in the data across different studies [7, 10]. A systematic review by Xu [26] reported the centre of the anteromedial (AM) bundle at 24.2% and 21.6%. However, limited literature is available regarding optimal positioning within the I.D.E.A.L. zone. A three‐dimensional CT (3D CT) simulation study by Yin [28] calculated the ideal I.D.E.A.L. centre at 28.4% and 22.2%. The I.D.E.A.L. tunnel is clinically described as a ‘high AM tunnel’ position, reflecting a location slightly superior to the classical AM centre. When this coordinate was adopted as the reference standard for dispersion analysis, no significant differences were observed between the two groups, which indicated that the femoral tunnel could be correctly positioned with either instrument. However, a distinct posterior positioning trend was observed with the modified locator (as evident in scatter plots), which is known to biomechanically favour reduced graft tension during knee motion [14, 15].

The traditional femoral locator was modified in this study by extending its tip to improve conformity with the posterior wall of the lateral femoral condyle. In actual surgical practice, the femoral posterior wall is used as a critical anatomical reference for tunnel positioning, and precise contact between the device tip and the posterior wall is considered essential for accurate tunnel preparation. Inadequate contact may lead to anterior deviation of the tunnel centre, thereby compromising positioning accuracy. It was observed that even when the traditional locator appeared to maintain sufficient posterior wall contact intraoperatively—and tunnel placement was judged to be satisfactory—postoperative CT scans occasionally revealed anteriorly displaced tunnels located outside the ideal I.D.E.A.L. zone. This discrepancy was attributed to anatomical variations in the morphology of the posterior edge of the lateral femoral condyle. In patients with an abrupt (nearly vertical) transition of the posterior condylar wall, traditional devices were found to achieve reliable contact, allowing for accurate tunnel placement. However, in cases with a gradual curvilinear transition, the short tip of the conventional device was often falsely registered against the oblique bony surface instead of the true posterior wall, resulting in anterior malposition of the tunnel.

To address this limitation, the locator was modified with these key design improvements: an elongated tip with a curvilinear surface that better matches variable condylar anatomy, ensuring true posterior wall contact rather than lateral wall abutment. This modification proved particularly advantageous during guide pin insertion at maximal knee flexion (100°–120°), where traditional instruments tend to lose reliable posterior contact. In contrast, tactile feedback from the posterior wall was consistently perceived with the modified locator. The enhanced tactile feedback of the new design was shown to maintain consistent posterior wall registration throughout extreme flexion, allowing for more intuitive and precise guidewire placement.

Our study has several limitations worth noting. First, all surgical procedures were performed by a single highly experienced surgeon, which likely minimised technical errors and judgement variability; results may differ when less‐experienced surgeons utilise these tools. Second, while tunnel centre position can vary with tunnel diameter, we were unable to perform tunnel zone analysis due to the lack of established I.D.E.A.L. zone parameters in the literature. Third, this study focused solely on radiographic tunnel position analysis without comparing functional outcomes between groups. To address these limitations, we plan to conduct a prospective comparative study incorporating: (1) multisurgeon participation across experience levels, (2) volumetric tunnel zone analysis, and (3) comprehensive functional assessments including patient‐reported outcomes and biomechanical testing.

## CONCLUSION

The modified locator facilitates femoral tunnel placement closer to the posterior wall, which promotes the biomechanical conditions required for optimal graft isometry and low tension.

## AUTHOR CONTRIBUTIONS

Hua Zhang designed this new locator. Pei Zhao, Shixin Nie and Hua Zhang have ideated the study and established the study design. Zhi Chen, Chengjian Wu and Jiajun Lin have collected the data. Pei Zhao and Shixin Nie performed the data analysis, data interpretation and prepared the manuscript. Chengjie Lian and Hua Zhang had substantially edited the draft.The authors read and approved the final manuscript.

## CONFLICT OF INTEREST STATEMENT

The authors declare that they have no conflict of interest.

## ETHICS STATEMENT

The study was conducted in accordance with the Declaration of Helsinki, and the protocol was approved by the Ethics Committee of the Fujian Medical University Union Hospital (approval number [2025KY686]).

## Data Availability

The data supporting this study's findings are available from the corresponding author upon reasonable request.

## References

[1] Ahn JH , Choi SH , Wang JH , Yoo JC , Yim HS , Chang MJ . Outcomes and second‐look arthroscopic evaluation after double‐bundle anterior cruciate ligament reconstruction with use of a single tibial tunnel. J Bone Jt Surg. 2011;93:1865–1872. 10.2106/JBJS.K.00136 22012523

[2] Bernard M , Hertel P , Hornung H , Cierpinski T . Femoral insertion of the ACL. Radiographic quadrant method. Am J Knee Surg. 1997;10:14–21. 9051173

[3] de Abreu‐e‐Silva GM , de Oliveira MHGCN , Maranhão GS , Deligne LMC , Pfeilsticker RM , Novais ENV , et al. Three‐dimensional computed tomography evaluation of anterior cruciate ligament footprint for anatomic single‐bundle reconstruction. Knee Surg Sports Traumatol Arthrosc. 2015;23:770–776. 10.1007/s00167-013-2703-9 24146049

[4] Desai N , Alentorn‐Geli E , van Eck CF , Musahl V , Fu FH , Karlsson J , et al. A systematic review of single‐ versus double‐bundle ACL reconstruction using the anatomic anterior cruciate ligament reconstruction scoring checklist. Knee Surg Sports Traumatol Arthrosc. 2016;24:862–872. 10.1007/s00167-014-3393-7 25344803

[5] Forsythe B , Kopf S , Wong AK , Martins CA , Anderst W , Tashman S , et al. The location of femoral and tibial tunnels in anatomic double‐bundle anterior cruciate ligament reconstruction analyzed by three‐dimensional computed tomography models. J Bone Joint Surg Am. 2010;92:1418–1426. 10.2106/JBJS.I.00654 20516317

[6] Irrgang JJ , Tashman S , Patterson CG , Musahl V , West R , Oostdyk A , et al. Anatomic single vs. double‐bundle ACL reconstruction: a randomized clinical trial‐Part 1: clinical outcomes. Knee Surg Sports Traumatol Arthrosc. 2021;29:2665–2675. 10.1007/s00167-021-06585-w PMC8298248 33970295

[7] Kim DH , Lim W , Cho S , Lim C , Jo S . Reliability of 3‐dimensional computed tomography for application of the bernard quadrant method in femoral tunnel position evaluation after anatomic anterior cruciate ligament reconstruction. Arthrosc ‐ J Arthrosc Relat Surg. 2016;32:1660–1666. 10.1016/j.arthro.2016.01.043 27090722

[8] Lee JK , Lee S , Seong SC , Lee MC . Anatomy of the anterior cruciate ligament insertion sites: comparison of plain radiography and three‐dimensional computed tomographic imaging to anatomic dissection. Knee Surg Sports Traumatol Arthrosc. 2015;23:2297–2305. 10.1007/s00167-014-3041-2 24817108

[9] Lertwanich P , Martins CAQ , Asai S , Ingham SJM , Smolinski P , Fu FH . Anterior cruciate ligament tunnel position measurement reliability on 3‐dimensional reconstructed computed tomography. Arthrosc ‐ J Arthrosc Relat Surg. 2011;27:391–398. 10.1016/j.arthro.2010.08.018 21126846

[10] Li J , Yang J , Xu Z , Wang W . Comparison of the quadrant method measuring four points and bernard method in femoral tunnel position evaluation on 3‐dimensional reconstructed computed tomography after anatomical single‐bundle anterior cruciate ligament reconstruction. BMC Musculoskelet Disord. 2024;25:558. 10.1186/s12891-024-07678-6 PMC11256444 39020301

[11] Li X , Lu J , Su J , Li H , Liu X , Ding R . High flexion femoral side remnant preservation positioning technique: a new method for positioning the femoral tunnel in anterior cruciate ligament reconstruction. J Orthop Surg. 2024;19:189. 10.1186/s13018-024-04670-7 PMC10949667 38500214

[12] Lubowitz JH . Anatomic ACL reconstruction produces greater graft length change during knee range‐of‐motion than transtibial technique. Knee Surg Sports Traumatol Arthrosc. 2014;22:1190–1195. 10.1007/s00167-013-2694-6 24077671

[13] Mutsuzaki H , Kinugasa T . Anatomical single‐bundle anterior cruciate ligament reconstruction using a calcium phosphate‐hybridized tendon graft with more than an average of 5 years of follow‐up: a follow‐up study of a randomized controlled trial. J Clin Med. 2023;12:4437. 10.3390/jcm12134437 PMC10342507 37445472

[14] Pearle AD , McAllister D , Howell SM . Rationale for strategic graft placement in anterior cruciate ligament reconstruction: I.D.E.A.L. femoral tunnel position. Am J Orthop (Belle Mead NJ). 2015;44:253–258. 26046994

[15] Pearle AD , Shannon FJ , Granchi C , Wickiewicz TL , Warren RF . Comparison of 3‐dimensional obliquity and anisometric characteristics of anterior cruciate ligament graft positions using surgical navigation. Am J Sports Med. 2008;36:1534–1541. 10.1177/0363546508315536 18390491

[16] Pietrini SD , Ziegler CG , Anderson CJ , Wijdicks CA , Westerhaus BD , Johansen S , et al. Radiographic landmarks for tunnel positioning in double‐bundle ACL reconstructions. Knee Surg Sports Traumatol Arthrosc. 2011;19:792–800. 10.1007/s00167-010-1372-1 21222103

[17] Rahr‐Wagner L , Thillemann TM , Pedersen AB , Lind MC . Increased risk of revision after anteromedial compared with transtibial drilling of the femoral tunnel during primary anterior cruciate ligament reconstruction: results from the Danish Knee Ligament Reconstruction Register. Arthrosc ‐ J Arthrosc Relat Surg. 2013;29:98–105. 10.1016/j.arthro.2012.09.009 23276417

[18] Samitier G , Marcano AI , Alentorn‐Geli E , Cugat R , Farmer KW , Moser MW . Failure of anterior cruciate ligament reconstruction. Arch Bone Jt Surg. 2015;3:220–240. PMC4628627 26550585

[19] Takahashi M , Doi M , Abe M , Suzuki D , Nagano A . Anatomical study of the femoral and tibial insertions of the anteromedial and posterolateral bundles of human anterior cruciate ligament. Am J Sports Med. 2006;34:787–792. 10.1177/0363546505282625 16452272

[20] Tsukada H , Ishibashi Y , Tsuda E , Fukuda A , Toh S . Anatomical analysis of the anterior cruciate ligament femoral and tibial footprints. J Orthop Sci. 2008;13:122–129. 10.1007/s00776-007-1203-5 18392916

[21] van Eck CF , Lesniak BP , Schreiber VM , Fu FH . Anatomic single‐ and double‐bundle anterior cruciate ligament reconstruction flowchart. Arthrosc ‐ J Arthrosc Relat Surg. 2010;26:258–268. 10.1016/j.arthro.2009.07.027 20141990

[22] Vermeijden HD , Yang XA , van der List JP , DiFelice GS , Rademakers MV , Kerkhoffs GMMJ . Trauma and femoral tunnel position are the most common failure modes of anterior cruciate ligament reconstruction: a systematic review. Knee Surg Sports Traumatol Arthrosc. 2020;28:3666–3675. 10.1007/s00167-020-06160-9 32691095

[23] Wang F , Wang G , Li Y , Li H , Shi Q , Li L . [Comparative study of I.D.E.A.L. technique and transtibial technique in anterior cruciate ligament reconstruction]. Zhongguo Xiu Fu Chong Jian Wai Ke Za Zhi. 2024;38:987–994.

[24] Webster KE , Feller JA . Exploring the high reinjury rate in younger patients undergoing anterior cruciate ligament reconstruction. Am J Sports Med. 2016;44:2827–2832. 10.1177/0363546516651845 27390346

[25] Wiggins AJ , Grandhi RK , Schneider DK , Stanfield D , Webster KE , Myer GD . Risk of secondary injury in younger athletes after anterior cruciate ligament reconstruction: a systematic review and meta‐analysis. Am J Sports Med. 2016;44:1861–1876. 10.1177/0363546515621554 PMC5501245 26772611

[26] Xu H , Zhang C , Zhang Q , Du T , Ding M , Wang Y , et al. A systematic review of anterior cruciate ligament femoral footprint location evaluated by quadrant method for single‐bundle and double‐bundle anatomic reconstruction. Arthrosc ‐ J Arthrosc Relat Surg. 2016;32:1724–1734. 10.1016/j.arthro.2016.01.065 27140814

[27] Yasuda K , van Eck CF , Hoshino Y , Fu FH , Tashman S . Anatomic single‐ and double‐bundle anterior cruciate ligament reconstruction, part 1: Basic science. Am J Sports Med. 2011;39:1789–1800. 10.1177/0363546511402659 21596902

[28] Yin L , Liao D , Xie Q , Liu J , Deng B . Characteristics of the femoral tunnel of anatomical and isometric single bundle anterior cruciate ligament reconstruction: a modeling analysis based on quadrant method and anatomical landmarks. J Orthop Surg. 2024;19:822. 10.1186/s13018-024-05306-6 PMC11616337 39633412

[29] Ziegler CG , DePhillipo NN , Kennedy MI , Dekker TJ , Dornan GJ , LaPrade RF . Beighton score, tibial slope, tibial subluxation, quadriceps circumference difference, and family history are risk factors for anterior cruciate ligament graft failure: a retrospective comparison of primary and revision anterior cruciate ligament reconstructions. Arthrosc ‐ J Arthrosc Relat Surg. 2021;37:195–205. 10.1016/j.arthro.2020.08.031 32911007

